# Recent advances in targeted therapy of pulmonary fungal infections with a focus on inhaled opelconazole (PC945): a systematic review

**DOI:** 10.22034/cmm.2026.345519.1746

**Published:** 2026-02-01

**Authors:** Nooshin Ahmadvand, Hossein Mohammad Taghi Fam, Iman Haghani, Nazanin Rahmanian, Mohammad Dodangeh Balakhani

**Affiliations:** 1 Department of Infectious Diseases, Faculty of Medicine, Guilan University of Medical Sciences, Rasht, Iran; 2 Shahid Beheshti Clinical Research Development Unit, Shahid Beheshti Hospital, Bandar-e Anzali, Guilan University of Medical Sciences, Rasht, Iran; 3 Invasive Fungi Research Center, Communicable Diseases Institute, Mazandaran University of Medical Sciences, Sari, Iran; 4 Department of Medical Mycology, School of Medicine, Mazandaran University of Medical Sciences, Sari, Iran; 5 Anesthesiology Research Center, Department of Anesthesiology, Alzahra Hospital, Guilan University of Medical Sciences, Rasht, Iran; 6 Department of Pulmonology, Faculty of Medicine, Guilan University of Medical Sciences, Rasht, Iran

**Keywords:** Antifungal Agents, Aspergillosis, Azoles, Fungal Infections, Opelconazole, Pulmonary

## Abstract

**Background and Purpose:**

Recent advancements in biomedical science have introduced advanced triazole agents for the targeted treatment of pulmonary diseases, including various respiratory fungal infections. Opelconazole (PC945) is an innovative triazole-based antifungal compound that functions as a potent inhibitor of the CYP51 enzyme. It is strategically engineered for inhalation administration to achieve elevated local concentrations within the lungs while minimizing systemic exposure. *In vitro* studies have demonstrated the exceptional antifungal potency of opelconazole against various fungal strains, including antifungal drug-resistant fungal species. Furthermore, in vivo experiments using murine models of invasive pulmonary aspergillosis and candidiasis have revealed the superior efficacy of inhaled opelconazole, compared to conventional systemic antifungal therapies.

**Materials and Methods:**

A systematic search was conducted in ScienceDirect, ClinicalTrials.gov, PubMed, and Scopus for studies published up to May 2025 using the terms "opelconazole" OR "PC945" OR "Pulmocide" OR "inhaled opelconazole". Inclusion criteria were studies reporting on opelconazole for human pulmonary fungal infections with clinical or mycological outcomes. Study selection, data extraction, and synthesis were conducted in accordance with the PRISMA guidelines.

**Results:**

Among 141 identified records, 10 studies met the inclusion criteria. Most studies focused on invasive aspergillosis. Based on the findings, inhaled opelconazole demonstrated favorable efficacy, reducing fungal burden and improving clinical outcomes, with a generally good safety profile and minimal adverse events.

**Conclusion:**

Inhaled opelconazole is a promising, well-tolerated option for pulmonary fungal infections, including those resistant to conventional therapy. According to the study results, this agent could be an effective treatment option for patients who do not respond to or cannot tolerate other treatments. However, larger randomized controlled trials are needed to confirm these results.

## Introduction

Fungal-associated pulmonary infections are among the most important causes of infections in hospitalized patients worldwide [1, 2]. They affect immunosuppressed patients and critically ill patients who undergo mechanical ventilation in intensive care units, regardless of the income level of the country [3, 4]. Despite the use of antifungal prophylaxis, mortality due to aspergillosis remains high among these patients, leading to increased systemic antibiotic consumption and prolonged stay in the intensive care unit [5, 6]. Chronic pulmonary *Aspergillus* infection can result in progressive lung destruction, necessitating long-term management primarily through the administration of oral azole antifungal therapy [7, 8]. Significant efforts have been made to manage respiratory fungal infections by enhancing our understanding of the underlying mechanisms and developing more effective treatment options for invasive infections.

As a mainstay of treatment, the administration of medications, including azoles (intravenously and orally) and echinocandins or amphotericin B (intravenously), has been shown to be effective in the treatment of pulmonary aspergillosis. Further work is required to determine whether combining azoles with other antifungals yields better clinical outcomes [9].

However, despite progress in the treatment of lung fungal infections, several important issues contribute to the failure of existing treatment modalities, which are mainly based on the systemic administration of antifungal medications.

Most antifungal medications, in various forms/doses, can cause toxic effects on specific organs and tissues, as well as adverse reactions, such as severe allergic reactions, liver toxicity, and electrolyte imbalances, like potassium and magnesium loss [10-12]. Although azole antifungal medications are potent inhibitors of cytochrome P450 enzymes, they pose a significant risk of drug-drug interactions with other co-administered drug classes [13]. Furthermore, attaining locally effective concentrations of intravenously or orally administered antifungal agents for the eradication of the pathogenic organism may be associated with elevated systemic exposure, thereby endangering the safety profile of these therapeutic agents [14, 15].

Hence, over the past few decades, extensive research has been conducted to develop nebulized delivery systems for antifungal therapeutics, aiming to maximize their targeted effects on fungal cells/tissues while minimizing adverse effects. Nebulized administration of antifungal agents can enable greater localized exposure into the pulmonary epithelial lining fluid, compared to intravenous infusion [16]. This approach offers the potential to improve therapeutic outcomes and reduce the risks associated with systemic administration.

### Opelconazole

Opelconazole is an antifungal azole specifically designed for the treatment and prophylaxis of bronchopulmonary infections through inhaled delivery [17]. Opelconazole (Figure 1), while sharing core structural features with posaconazole, such as a central triazole ring and a difluorophenyl group attached to a dioxolane ring, possesses a distinct benzamide substituent, contributing to its high potency as an ergosterol synthesis inhibitor in *Aspergillus fumigatus*, with an IC_50_ (cell-based ergosterol production assay) of 6.9 nM, 14-fold and 2.6-fold more potent than voriconazole and posaconazole, respectively [18, 19].

**Figure 1:**
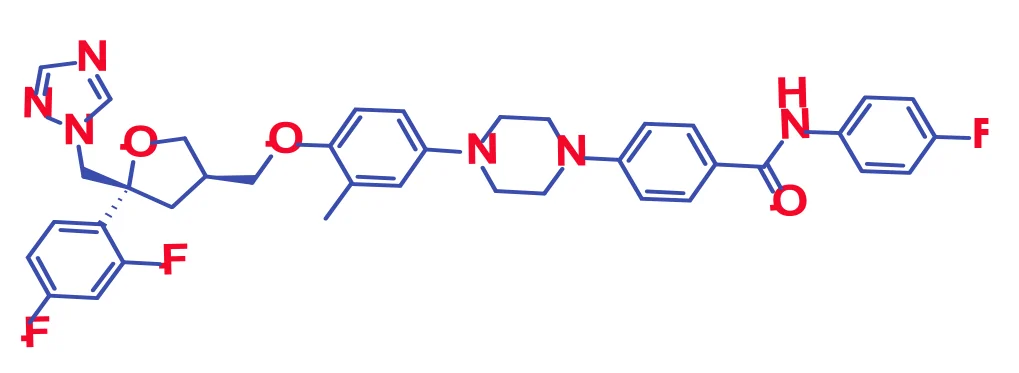
Structural formula of opelconazole (PC-945). Nebulized opelconazole potentially exhibits long-term retention in alveolar cells with high protein binding and minimal oral availability [17]. Opelconazole exhibited significant efficacy against a range of azole-resistant and azole-sensitive strains of *Aspergillus fumigatus* and the emerging yeast *Candida auris* [20-22]. Furthermore, Opelconazole is in phase III of clinical trials (NCT05238116) as of 2022 [23].

### Mechanism of action and resistance of opelconazole

Opelconazole, like other triazole antifungals, has an inhibitory effect on the enzyme CYP51A1 (lanosterol 14α-demethylase), which is a fungal cytochrome P450-dependent enzyme [19, 24]. This enzyme is responsible for the conversion of lanosterol to ergosterol, an essential sterol in fungal cell membranes. Inhibition of this enzyme disrupts ergosterol synthesis, resulting in the accumulation of abnormal 14α-methylsterol metabolites, which increase the permeability of the fungal cell membrane and impair its function. This stops the growth of the fungus and, at high doses, can lead to cell death (fungicidal effect).

Therefore, the basic mechanism of action of opelconazole is the reduction of ergosterol in the fungal cell membrane, which disrupts the function and structure, and thus prevents the growth and spread of the fungus. In a preliminary study, the local minimum inhibitory concentrations (MICs) of opelconazole, fluconazole, posaconazole, and voriconazole were compared against Candida auris. In the aforementioned study, the researchers claimed that there was a reasonable correlation between the MICs of opelconazole and those of posaconazole/voriconazole. However, the MIC of opelconazole showed no correlation with ERG11 mutation status [22]. It seems that efflux pumps play a significant role in the multidrug resistance mechanisms of *C. auris*. This indicates that the resistance mechanism of the antifungal agent opelconazole is similar to those of posaconazole and voriconazole in isolates with poor susceptibility to opelconazole, although an analysis of the cross-resistance mechanism is warranted. However, the specific mechanisms responsible for the elevated MIC remain unexplored.

### Combination of opelconazole with other antifungal medications

Combination treatment approaches with topical opelconazole and systemic azoles led to synergistic interaction, with potency improved as a monotherapy against triazole-susceptible and resistant respiratory *A. fumigatus* invasion [25-27]. Recently, a study was conducted to assess whether combination therapy with opelconazole, together with voriconazole or posaconazole, is effective against *A. fumigatus* infection [28]. In the above-mentioned study, the researchers claimed that topical combination therapy with opelconazole led to widespread synergistic effects in the lungs of neutropenic immunocompromised mice and in a human alveolus bilayer model *in vitro*. Opelconazole administered in the upper chamber exhibited partial inhibition of fungal growth. In contrast, the combined regimen employing opelconazole in the upper chamber and posaconazole in the lower chamber demonstrated a potent inhibitory effect on fungal growth. These beneficial effects were also reported when opelconazole was combined with voriconazole and itraconazole. Moreover, 83% of mice treated with a combination of opelconazole (intranasally) and posaconazole (orally) survived until day 7. However, notably, repeated preventive treatment with opelconazole alone indicated superior effects, probably due to the accumulation of the opelconazole agent in lung tissue. Despite advancements in systemic antifungal therapies, there are significant limitations to these approaches, including systemic toxicity, potential drug interactions, and the emergence of drug-resistant strains. These challenges underscore the need for targeted therapies that can effectively eliminate the infectious agent and ensure that antifungal agents reach adequate concentrations directly at the site of infection in the lungs.

Therefore, this systematic review aimed to thoroughly evaluate clinical trials and observational studies regarding the efficacy, safety, and therapeutic use of inhaled opelconazole for the management of pulmonary fungal infections.

## Materials and Methods

### Search strategy

A systematic search was conducted in the electronic databases ScienceDirect, ClinicalTrials.gov, PubMed, and Scopus for articles published up to the end of May 2025. In addition, relevant studies were identified through manual search of reference lists and supplementary sources. The search terms used were "opelconazole" OR "PC945" OR "Pulmocide" OR "inhaled opelconazole" AND "aspergillosis" OR "pulmonary fungal infection" AND "antifungal" OR "triazole". The search strategy was devised following the guidelines outlined in the Preferred Reporting Items for Systematic Reviews (PRISMA) framework [29]. This systematic review was conducted in accordance with PRISMA guidelines.

### Inclusion criteria

The inclusion criteria consisted of studies investigating the use of opelconazole for the treatment of human pulmonary fungal infections and reporting relevant clinical outcomes, such as clinical response, mycological response, mortality, or adverse events. *In vitro* and animal studies were not included in this systematic review and were discussed only as supportive background information.

### Exclusion criteria

Studies published in languages other than English, review articles, editorials, book chapters, short surveys, notes, letters, duplicate publications, or studies involving human subjects were excluded from this review. Duplicate articles were identified and removed using citation management software.

### Study selection

Articles that appeared to meet the inclusion criteria or for which there was insufficient information to make a decision were retrieved for full-text review. The full texts of these articles were then assessed based on the inclusion criteria. The following information was extracted from the studies: study characteristics, patient characteristics, details of the interventions, and outcomes.

### Data extraction

Data extraction was performed by one reviewer and verified by a second reviewer to ensure accuracy. A quantitative meta-analysis was not feasible due to the limited number of studies. Instead, the findings of the included studies were combined narratively. The quality of the studies included was assessed using appropriate tools based on the study design. Clinical trials were evaluated using the Cochrane Risk of Bias tool, while case reports were assessed using the case report guidelines.

## Results

### Study Flow

An initial search of electronic databases yielded 141 records. After the removal of 13 duplicate records, 128 records were screened based on their titles and abstracts. Among these, 88 records were excluded due to irrelevance or failure to meet the predefined inclusion criteria. Full texts of the remaining 40 articles were retrieved and assessed for eligibility. Among these, 30 reports were excluded, and 10 studies met the inclusion criteria and were included in the systematic review (Figure 2). Most included studies were case reports.

**Figure 2:**
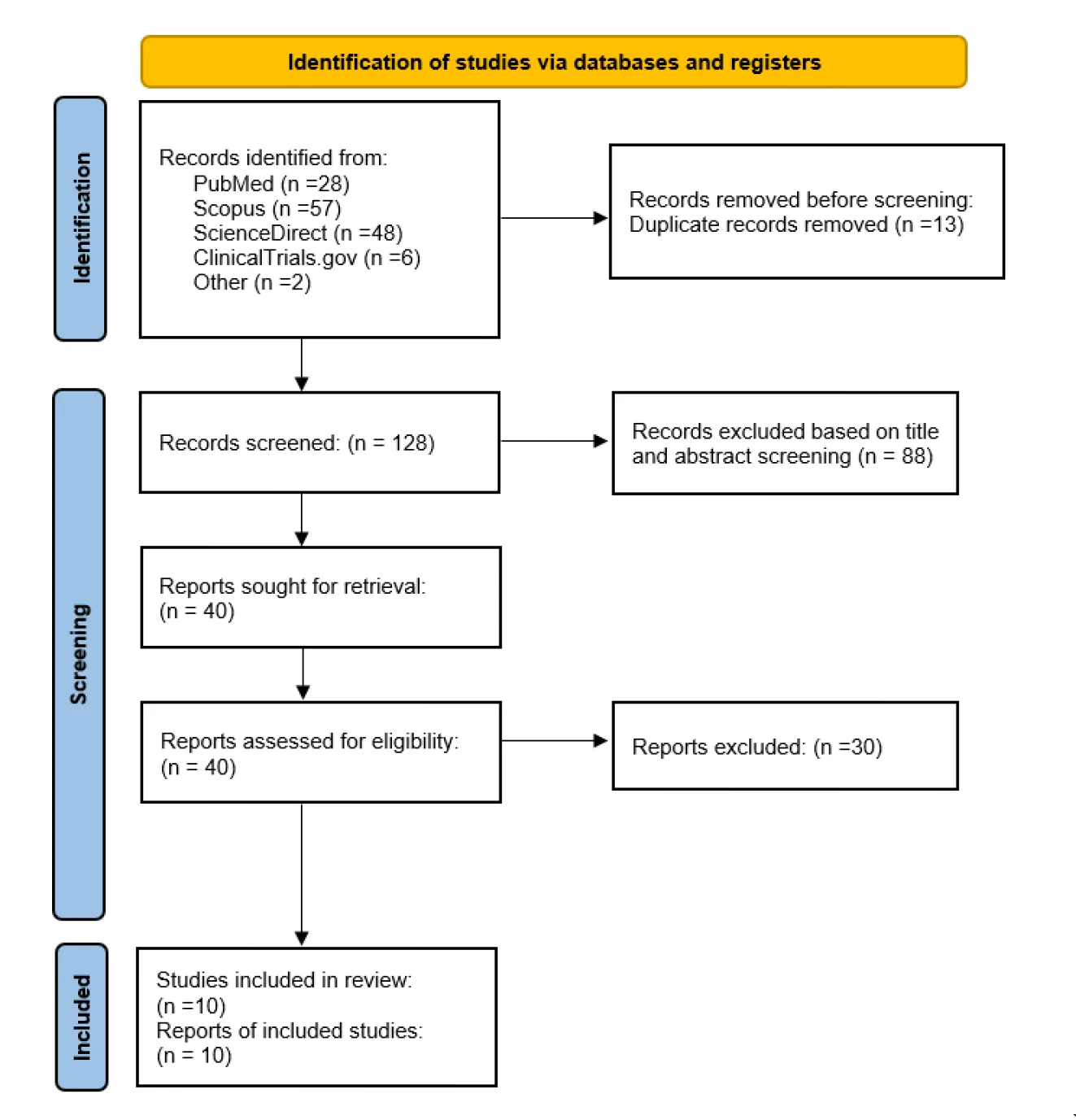
Study flow chart based on Preferred Reporting Items for Systematic Reviews. A total of 141 records were identified from PubMed (n=28), Scopus (n=57), ScienceDirect (n=48), ClinicalTrials.gov (n=6), and other sources (n=2). After the removal of 13 duplicate records, 128 records were screened. Among these, 88 records were excluded based on title and abstract screening and predefined eligibility criteria, and 40 reports were sought for retrieval. All 40 reports were assessed for eligibility, of which 30 were excluded, and 10 studies were included in the final review.

### Study characteristics

Characteristics of the 10 studies included in this review are summarized in Tables 1 and 2. Diagnostic methods varied across studies and included culture, microscopy, bronchoscopy, serological markers (e.g., galactomannan and beta-D-glucan), molecular assays, and imaging techniques, such as computed tomography scans [30-33]. These studies were published between 2017 and 2025, with most of them originating from the UK, although ongoing research is also being conducted in the US and other countries.

**Table 1:** Characteristics of studies included in the review

Variables	Pagani et al. 2020 [30]	Cass et al. 2021[31]	Singh et al. 2022 [33]	Nwankwo et al. 2025 [32]
Study type	Case Report	Original	Case Report	Case Report
Country	U. K	U. K	U. K	U. K
Patients	2	29	1	1
Male / Female	1/1	13/16	0/1	0/1
Age means (years)	38	39.8	30	54
Disease	Invasive Aspergillus infection of the respiratory tract	-	Pseudomembranous, ulcerative Aspergillus tracheobronchitis (ATB)	severe asthma and uncontrolled bronchiectasis ABPA
Treatment duration with Opelconazole	3 Month	Single dose/1 week	6 Week	long-term treatment
Treatment	PC945, posaconazole, terbinaﬁne	PC945	PC945, Anidulafungin, Liposomal Amphotericin, Voriconazole, Isavuconazole, Itraconazole, etc.	Opelconazole, corticosteroids, mepolizumab, and caspofungin
Dosage use	5 mg once or twice daily	0.5, 2, 5, & 10 mg once daily	Not reported	14.8 & 29.6 mg
Improvement	Improvement after 2 weeks and complete dissolution after 8 weeks	No significant adverse events were reported in healthy subjects and subjects with mild asthma.	Successful treatment of pseudomembranous Aspergillus tracheobronchitis with PC945	Clinical, mycologic, and radiologic responses within 3 months; serologic responses required >6 months
	Bronchoscopy showed significant shrinkage of the fungal mass after 2 weeks. One month later, the anastomosis appeared normal.			
Adverse Events	No significant adverse events were reported.	Treatment - emergent adverse events were mild/moderate and resolved before the study ended.	None directly reported	Transient chest/wheeze during nebulization with initial doses of 29.6 mg BID

TB: Aspergillus Tracheobronchitis, BID: Twice a Day

**Table 2:** Characteristics of clinical trials evaluating inhaled opelconazole

Variables\ Phase	Phase 1	Phase 2	Phase 2	Phase 2	Phase 3	Phase 3
**ClinicalTrials.gov ID**	NCT02715570[38]	NCT03745196[39]	NCT03870841[40]	NCT05037851[41]	NCT03905447[42]	NCT05238116 [23]
**Status**	Completed	Terminated early as a result of the COVID-19 outbreak	Terminated early as a result of the COVID-19 outbreak	Completed	Terminated early as a result of the COVID-19 outbreak	Recruiting
**locations**	United Kingdom	United Kingdom	United Kingdom	United States-Canada	United Kingdom	United States, Argentina, Australia, Austria, Belgium, Brazil, Canada, Chile, Colombia, France, Germany, Greece, India, Italy, Korea, Republic of, Spain, Taiwan, Thailand, United Kingdom
Study Start (Actual)	2017-10-23	2018-11-15	2019-04-03	2021-11-19	2019-09-17	2022-06-14
**Study Completion (Actual)^1^**	2018-04-27	2020-06-01	2020-06-01	2023-11-13	2020-06-01	2026-04-30
**Enrollment (Actual)**	29	13	4	102	2	123
**Conditions**	Aspergillosis	Asthma, Respiratory Candidiasis, Respiratory Aspergillosis, COPD Bronchiectasis	Aspergillosis Cystic Fibrosis	Pulmonary Aspergillosis	Aspergillosis Lung Transplant Infection	Refractory IPA
**Brief Summary**	Investigate the safety, tolerability, and pharmacokinetics of single and repeat doses of PC945.	Testing the effects of PC945 in people with asthma or other chronic respiratory diseases whose lungs are infected with *Aspergillus fungi* and *Candida* yeasts	Testing the effects of PC945 in people with cystic fibrosis whose lungs are infected with the fungus *A. fumigatus*	Evaluate the safety and tolerability of opelconazole for the prevention of fungal *Aspergillus* infections in the lung in participants who have received a lung transplant.	Testing the effects of preventive treatment with the experimental drug PC945 in lung transplant recipients whose lungs are infected with the fungus *A. fumigatus*	To assess the safety and efficacy of nebulized PC945 in combination with systemic antifungal therapy for the treatment of refractory IPA

1 Trial completion dates are reported as actual based on the information available at the time of data collection. Data accessed on May 2025.

COPD: Chronic Obstructive Pulmonary Disease, IPA: Invasive Pulmonary Aspergillosis

Among the studies, there were case reports, one was original research, six were clinical trials, and one was an interventional study. The number of cases and sample sizes varied, ranging from 1 to 123 participants. The most frequently studied type of pulmonary fungal infection was invasive aspergillosis. Dose of inhaled opelconazole differed across the studies, varying from 0.5 mg to 29.6 mg, administered once daily. Treatment duration also varied, ranging from a single dose to several years.

### Clinical and mycological outcomes

Clinical trials consistently demonstrate a reduction in fungal burden and an improvement in respiratory function among patients treated with opelconazole for invasive aspergillosis and other pulmonary fungal infections. For instance, Pagani et al. in their study reported significant improvement after just two weeks of treatment with opelconazole in patients suffering from invasive *Aspergillus* infections of the respiratory tract [30].

In one case study of fungal bronchial anastomotic infection caused by *A. fumigatus* after lung transplantation treated with combination therapy (opelconazole, posaconazole, and terbinafine), a fungal complex susceptible to systemic triazoles, echinocandins, and amphotericin B was identified in bronchoalveolar lavage. Addition of terbinafine did not prevent the progression. However, administration of nebulized opelconazole (5 mg once daily) confirmed improvement after 2 weeks of treatment, showing significant shrinkage of the fungal mass. Moreover, the anastomosis appeared normal one month later [30].

Another case study reported a 29-year-old woman with a bronchial anastomotic infection due to *A. fumigatus* after bilateral lung transplantation. According to the aforementioned study, previous treatment with inhaled amphotericin B, oral terbinafine, and posaconazole had resulted in worsening of fungal hyphae attached to anastomotic sutures and endobronchial erythema. After two weeks of treatment with inhaled opelconazole, in addition to continued oral terbinafine and posaconazole, bronchoscopy results showed a reduction in the size of the anastomotic lesion [30].

In a third case report, a 30-year-old woman with concurrent systemic lupus erythematosus and hemophagocytic lymphohistiocytosis developed pseudomembranous *Aspergillus* tracheobronchitis infection. After failure to achieve a clinical response with a combination of liposomal amphotericin, isavuconazole, voriconazole, and caspofungin over 7 days, rescue therapy with inhaled opelconazole was initiated. Repeat bronchoscopy after treatment showed improved mucosal integrity and significant clearance of the infection [33].

Additionally, Nwankwo et al. [32] reported positive results from using opelconazole in patients with severe asthma and uncontrolled bronchiectasis due to allergic bronchopulmonary aspergillosis. Clinical, mycological, and radiologic responses were observed within 3 months, while serological responses required more than 6 months.

### Safety and adverse events

In general, opelconazole was well-tolerated, with adverse drug reactions (AEs) occurring rarely. Most AEs were mild, including symptoms such as cough, nausea, and sore throat. Serious AEs were infrequent and not definitively linked to the drug. Additionally, no significant changes in lung function parameters were observed. Inhalation administration of opelconazole was well-tolerated by subjects with mild asthma. However, some studies reported transient adverse events during nebulization, such as chest pain, wheezing, and irritation of the respiratory tract [30-32], particularly with initial doses of 29.6 mg twice daily.

## Discussion

### Interpretation of efficacy and clinical value

In this systematic review, an attempt was made to review the latest trends in targeted therapy of pulmonary fungal infections using inhaled opelconazole. The findings suggest that opelconazole has good potential in the treatment of pulmonary fungal infections, especially invasive aspergillosis. Most studies have shown a positive response to inhaled opelconazole in the management of pulmonary fungal infections. The included studies demonstrated clinical improvement, reduction in fungal burden, and good tolerability in patients treated with opelconazole.

The clinical cases highlight the real-world utility of this agent. For instance, treating active tracheobronchitis with anastomotic infection with azoles is challenging due to adverse drug reactions with calcineurin inhibitors. Systemic amphotericin B is generally avoided in lung transplant patients due to the high risk of nephrotoxicity. Furthermore, inhaled drug delivery is often problematic due to issues related to its poor tolerability.

Furthermore, the post-transplant bronchial anastomosis site experiences reduced blood flow, thereby limiting the ability of intravenously administered antifungal agents to effectively penetrate the site of infection. In these scenarios, inhaled opelconazole was better tolerated, with no systemic or local adverse effects, providing a viable alternative [34].

Moreover, the diagnosis of *Aspergillus* tracheobronchitis infection is often complicated, leading to late diagnosis and a high mortality rate. Bronchoscopy biopsy with microscopy, which demonstrates the presence of Aspergillus-like hyphae, remains the most sensitive diagnostic approach for monitoring such treatment success [33].

### Comparison with other antifungal agents and pharmacokinetics

The poor therapeutic response to systemic antifungals is believed to be due to poor local penetration, insufficient drug concentration at the site of infection, and pharmacokinetic variability. Currently available triazole compounds have serious limitations, including restrictions on route of administration and dosage, drug interactions, and treatment-limiting side effects.

In contrast, comparative studies have shown significant antifungal efficacy of topical opelconazole compared with voriconazole and posaconazole in *C. auris* isolates that are resistant to fluconazole [22]. Nebulized opelconazole produces strong synergistic effects when combined with systemic triazoles.

In other studies, pulmonary pharmacokinetic data for opelconazole in subjects with mild asthma have been reported to be like those for oral posaconazole in lung transplant patients, suggesting that absorption in pulmonary alveolar epithelial cells is a reproducible distribution characteristic of this drug in clinical trials [31, 35, 36].

### Mechanistic insights into safety and delivery advantages

Clinical investigations in healthy participants and patients with mild asthma demonstrated that opelconazole, delivered via inhalation, achieves significantly elevated lung concentrations while maintaining minimal systemic exposure, resulting in excellent tolerability and only mild, transient side effects. Pharmacokinetics in humans were dose-proportional, exhibiting linear behavior across the tested dosage range, indicating predictable and consistent drug absorption and elimination, contributing to a low potential for drug-drug interactions due to low systemic exposure. No significant lung function changes were reported, suggesting opelconazole offers targeted lung therapy with reduced risk of pulmonary side effects [31].

Current antifungal agents are typically administered systemically orally and have limitations, including unwanted systemic effects and inadequate drug concentration at the primary site of infection. The aqueous formulation of opelconazole, which is presented as a ready-to-use suspension compatible with commercially available nebulizers for effective drug delivery to the lungs, has the potential to circumvent these issues. Therefore, targeted treatment of pulmonary fungal infections with aerosolized triazoles, such as opelconazole, could avoid systemic toxicities.

Usage of the inhalation route to deliver the drug to the lungs has potential advantages over systemic therapies, including increased drug concentration at the site of infection and reduced systemic side effects. A discussion of safety concerns, along with regulatory and clinical aspects of the agent opelconazole, suggests the desirability of using the antifungal nebulized opelconazole in the treatment of invasive pulmonary aspergillosis and the emerging yeast C. auris [37].

### Limitations and future directions

This review has limitations that should be considered. The number of studies available on inhaled opelconazole is still limited. Case reports are inherently subject to a higher risk of bias. Doses of opelconazole, patient populations, and underlying conditions varied across studies. Most studies were conducted in a limited number of geographic regions, potentially affecting the generalizability of the findings. Some studies did not provide sufficient information about the study design and study outcomes.

Ongoing clinical trials are being conducted to address these limitations and further evaluate the effectiveness of inhaled opelconazole across diverse populations and disease states. For example, an ongoing trial (NCT05238116) is currently evaluating the effectiveness of opelconazole in combination with standard systemic antifungal therapy for the treatment of refractory invasive pulmonary aspergillosis, compared to standard therapy alone [23].

## Conclusion

Pulmonary fungal infections pose a substantial global disease burden, with annual mortality rates equivalent to those of HIV and tuberculosis. However, to date, insufficient attention has been devoted to engineering more advanced, clinically effective antifungals to combat the growing threat of multidrug resistance in various fungal pathogens. The primary challenge lies in developing novel clinical antifungals with maximal specific effects on fungal cells and minimal side effects on healthy cells. The fungi and their hosts are eukaryotes, which makes it challenging to identify unique targets for drugs. Furthermore, the emergence of resistant fungal strains is largely dependent on the occurrence of inadvertent adverse drug-drug interactions and toxicity, which can manifest as invasive pulmonary fungal infections.

Opelconazole, formulated as a ready-to-use aqueous suspension compatible with standard nebulizers, offers a promising treatment to address these concerns. Clinical trials have confirmed that the nebulized formulation of opelconazole exhibits a favorable pharmacokinetic and safety profile, accompanied by a prolonged pulmonary residence time and acceptable tolerability. Such potential heralds them as a novel class of triazoles for the development of antifungal drug delivery systems with exceptional potential for targeted therapy. To date, the obtained patient case histories are highly promising. Pulmocide Ltd. is currently focusing on the commercialization of opelconazole in collaboration with regulatory agencies, including the US Food and Drug Administration (FDA), to complete the clinical development of opelconazole in the treatment of invasive pulmonary aspergillosis. Therefore, it may now be appropriate to consider opelconazole as a potential inhaled antifungal treatment option.
